# Enhanced CO evolution for photocatalytic conversion of CO_2_ by H_2_O over Ca modified Ga_2_O_3_

**DOI:** 10.1038/s42004-020-00381-2

**Published:** 2020-10-09

**Authors:** Rui Pang, Kentaro Teramura, Masashige Morishita, Hiroyuki Asakura, Saburo Hosokawa, Tsunehiro Tanaka

**Affiliations:** 1 Department of Molecular Engineering, Graduate School of Engineering, Kyoto University, Kyotodaigaku Katsura, Nishikyo-ku, Kyoto 615-8510 Japan; 2 Element Strategy Initiative for Catalysts & Batteries (ESICB), Kyoto University, 1-30 Goryo-Ohara, Nishikyo-ku, Kyoto 615-8245 Japan

**Keywords:** Artificial photosynthesis, Photocatalysis, Heterogeneous catalysis, Devices for energy harvesting

## Abstract

Artificial photosynthesis is a desirable critical technology for the conversion of CO_2_ and H_2_O, which are abundant raw materials, into fuels and chemical feedstocks. Similar to plant photosynthesis, artificial photosynthesis can produce CO, CH_3_OH, CH_4_, and preferably higher hydrocarbons from CO_2_ using H_2_O as an electron donor and solar light. At present, only insufficient amounts of CO_2_-reduction products such as CO, CH_3_OH, and CH_4_ have been obtained using such a photocatalytic and photoelectrochemical conversion process. Here, we demonstrate that photocatalytic CO_2_ conversion with a Ag@Cr-decorated mixture of CaGa_4_O_7_-loaded Ga_2_O_3_ and the CaO photocatalyst leads to a satisfactory CO formation rate (>835 µmol h^−1^) and excellent selectivity toward CO evolution (95%), with O_2_ as the stoichiometric oxidation product of H_2_O. Our photocatalytic system can convert CO_2_ gas into CO at >1% CO_2_ conversion (>11531 ppm CO) at ambient temperatures and pressures.

## Abstract (web summary)

Photocatalytic CO_2_ reduction requires catalysts that are both active and selective. Here, a mixture of CaGa_4_O_7_-loaded Ga_2_O_3_ and CaO, decorated with Ag@Cr core-shell particles, delivers over 835 µmol h^−1^ of CO at >95 % selectivity.

## Introduction

Carbon dioxide (CO_2_) concentrations in the atmosphere have increased drastically over the past few centuries owing to the combustion of carbon-rich fossil fuels such as coal, oil, and natural gas. As a major anthropogenic greenhouse gas, these ever-increasing CO_2_ emissions are detrimental to the environment and will affect both ecosystems and the global climate^1^. Therefore, there is a critical requirement of mitigating CO_2_ emissions to achieve sustainable development. Since the pioneering work on the photocatalytic conversion of CO_2_ into formic acid (HCOOH) and methyl alcohol (CH_3_OH) over semiconductors reported by Halmann and Inoue et al.^2,3^, the photocatalytic conversion of CO_2_ into other valuable feedstocks at ambient temperatures and pressures has attracted considerable attention from the scientific community as a feasible strategy for CO_2_ storage and conversion^4–8^.

In general, the photocatalytic conversion of CO_2_ over an excited semiconductor-based catalyst involves three main steps. First, CO_2_ molecules are adsorbed on the photocatalyst surface^9–11^. Second, the photogenerated electrons react with the adsorbed CO_2_ species and protons (H^+^) to yield products such as carbon monoxide (CO), methane (CH_4_), CH_3_OH, and HCOOH. Among these possible reduction products, CO is one of the most useful because it is widely combined with H_2_ to provide synthetic gas for use in many chemical processes, such as methanol synthesis^12,13^ and the industrial Fischer–Tropsch process that produce various chemicals and synthetic fuels^14,15^. Third, the products are desorbed from the photocatalyst surface. However, as the H/H_2_ redox potential (−0.41 V vs. NHE at pH 7) is more positive than that for CO_2_/CO (−0.52 V vs. NHE at pH 7), the generation of H_2_ from H^+^ is preferable for the photocatalytic conversion of CO_2_ into CO, where H_2_O acts as the electron donor^16–18^. Moreover, because of the high thermodynamic stability of the linear CO_2_ molecule, the fixation and activation of CO_2_ are also immense challenges in the photocatalytic conversion of CO_2_ by H_2_O^4,19^. Thus, although various heterogeneous photocatalysts have been reported for the photocatalytic conversion of CO_2_ into CO with H_2_O as the electron donor^20–24^, the photocatalytic activity for CO evolution remains limited to a few micromoles, while the photocatalytic conversion rate of CO_2_ into CO is <0.15%.

Based on the processes involved in the photocatalytic conversion of CO_2_ described previously, we deduce that the photocatalytic activity of the photocatalyst for CO_2_ conversion can be improved by increasing CO_2_ adsorption, charge separation, and product desorption. Due to the fact that CO_2_ acts as a Lewis acid that bonds easily with Lewis bases^25^, many studies have focused on improving CO_2_ adsorption by modifying the photocatalyst surface with a CO_2_ adsorbent, such as NaOH^26^, amino groups^27^, and rare earth species^28^, to increase the photocatalytic activity and selectivity for CO_2_ conversion by H_2_O. Our group reported that modifying the photocatalyst surface with alkaline earth metals (e.g., Ca, Sr, and Ba) enhanced the conversion of CO_2_ and the selectivity toward CO evolution^29^. Moreover, we found that a Ag@Cr core/shell cocatalyst suppresses the backward reaction from CO and O_2_ to CO_2_, and enhances the adsorption of CO_2_, resulting in a highly selective photocatalytic CO_2_ conversion^30,31^.

In this study, we exploited the above techniques and successfully fabricated a Ag@Cr-decorated mixture of CaGa_4_O_7_-loaded Ga_2_O_3_ and CaO photocatalyst, which exhibits a high CO formation rate (>835 µmol h^−1^) per 0.5 g of catalyst, in addition to high selectivity toward CO evolution (>95%) with the stoichiometric production of O_2_ as the oxidation product of H_2_O during the photocatalytic conversion of CO_2_ by H_2_O. Approximately 1.2% of the CO_2_ in the gas phase was transformed into CO (11531 ppm) as a product. The results reported in this study represent almost an order of magnitude higher than most previously published results, as summarized in Supplementary Table 1.

## Results and discussion

### Photocatalytic reduction of CO_2_ by H_2_O

Table 1 shows the formation rates of CO, H_2_, and O_2_, selectivity toward CO evolution, and the balance between consumed electrons and holes over the bare Ga_2_O_3_, Ag-modified Ga_2_O_3_ (Ag/Ga_2_O_3_), Ag@Cr-modified Ga_2_O_3_ (Ag@Cr/Ga_2_O_3_), and Ag@Cr-modified Ca-loaded Ga_2_O_3_ (Ag@Cr/Ga_2_O_3__Ca) photocatalysts during the photocatalytic conversion of CO_2_ by H_2_O. No liquid products were detected in the reaction solutions in these photocatalytic systems, and H_2_, O_2_, and CO were detected as gaseous products. As no reduction products other than H_2_ and CO were generated, the selectivity toward CO evolution and the balance between the consumed electrons and holes were calculated as follows:

1 $${\mathrm{Selectivity}}\,{\mathrm{toward}}\,{\mathrm{CO}}\,{\mathrm{evolution}}\,\left( \% \right) = 2R_{{\mathrm{CO}}}/(2R_{{\mathrm{CO}}} + 2R_{{\mathrm{H}}2}) \times 100,$$

2 $${\mathrm{Consumed}}\,e^ - /h^ + = (2R_{{\mathrm{CO}}} + 2R_{{\mathrm{H}}2})/4R_{{\mathrm{O}}2},$$

**Table 1:** Photocatalytic conversions of CO_2_ by H_2_O using various photocatalysts.

Catalyst	Formation rates of products (µmol h^−1^)			Selec. toward CO (%)	Consumed *e*^−^/*h*^+^
	H_2_	O_2_	CO		
Bare Ga_2_O_3_	240.9	122.8	9.8	4	1.02
Ag/Ga_2_O_3_	248.3	172.7	102.1	29	1.01
Ag@Cr/Ga_2_O_3_	148.5	316.4	499.6	77	1.02
Ag@Cr/Ga_2_O_3__Ca	176.5	448.2	794.2	82	1.08

Photocatalyst powder: 0.5 g, reaction solution volume: 1.0 L, additive: 0.1 M NaHCO_3_, CO_2_ flow rate: 30 mL min^−1^, light source: 400-W high-pressure Hg lamp.

where *R*_CO_ and *R*_H2_ represent the formation rates of CO and H_2_, respectively. If H_2_O acts as an electron donor, the value of *e*^−^/*h*^+^ should be equal to 1.

We obtained stoichiometric amounts of H_2_ and CO as reduction products in addition to O_2_ as the oxidation product, indicating that H_2_O serves as the electron donor. Bare Ga_2_O_3_ exhibited a particularly low selectivity toward CO evolution (4%) as the electrons generated by charge transfer were not consumed in the reduction of CO_2_, but rather in the production of H_2_ from H^+^. Modifying Ga_2_O_3_ with a Ag cocatalyst enhanced the selectivity toward CO evolution (29%); however, this was not sufficient to obtain a selectivity >50%. In contrast, we succeeded in the selective photocatalytic conversion of CO_2_ by H_2_O over Ag@Cr/Ga_2_O_3_. A relatively high CO formation rate (499.6 µmol h^−1^) was achieved with 77% selectivity toward CO evolution. The photocatalytic reaction for the conversion of CO_2_ by H_2_O over Ag@Cr/Ga_2_O_3_ and Ag@Cr/Ga_2_O_3__Ca was carried out for at least four times, and errors in the product formation rates (H_2_, O_2_, and CO) were smaller than 5%. Controlling both, the bulk and surface of the photocatalysts, is highly important for achieving a considerably high CO formation rate and selectivity toward CO evolution. We found that the amount of Ca species significantly affected the H_2_ and CO formation rates (for the product formation rates and selectivity over various Ag@Cr/Ga_2_O_3__Ca photocatalysts see Supplementary Fig. 1). The formation rate of CO increased first and then decreased as the Ca content increased (Supplementary Fig. 1a–g). In contrast, the formation rate of H_2_ over the Ag–Cr/Ga_2_O_3__Ca_*x* samples increased monotonically with increasing amount of Ca species. The Ag–Cr/CaGa_4_O_7_ photocatalyst was only active for H_2_ evolution derived from water splitting (Supplementary Fig. 1h). The Ag@Cr/Ga_2_O_3__Ca photocatalyst exhibited the highest CO formation rate (794.2 µmol h^−1^), and the selectivity toward CO evolution was approximately 82%. Additionally, CO production from the photocatalytic conversion of CO_2_ after photoirradiation for 15 h over Ag@Cr/Ga_2_O_3__Ca was more stable than that over Ag@Cr/Ga_2_O_3_ (for the product formation rates for 15 h see Supplementary Fig. 2), which indicates that the presence of Ca species is not only beneficial for improving the photocatalytic activity and selectivity, but also for improving stability during the photocatalytic conversion of CO_2_ to CO.

Various control experiments were carried out to confirm the source of CO during the photocatalytic conversion of CO_2_ by H_2_O, the results of which are shown in Supplementary Fig. 3. We did not detect any appreciable amounts of products under dark conditions or in the absence of a photocatalyst. In addition, H_2_ was the main product formed when Ar gas was used instead of CO_2_ or in the absence of NaHCO_3_. The control experiments confirmed that the evolved CO originated from the CO_2_ gas introduced into the samples and not from carbon contaminants.

### Photocatalyst characterization

**Fig. 1:**
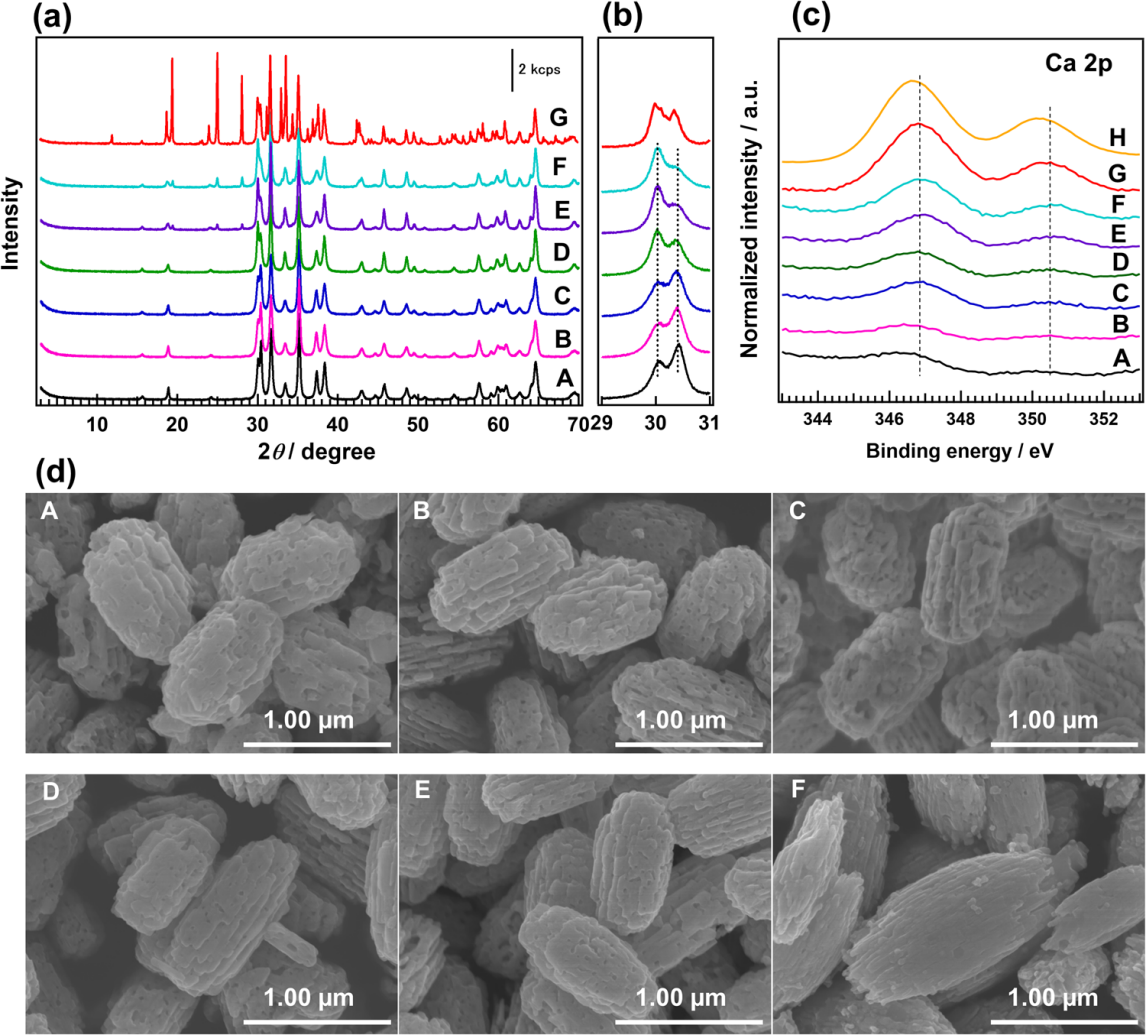
Photocatalyst characterization. **a** X-ray diffractograms; **b** enlarged X-ray diffractograms at 2*θ* = 29–31°; **c** Ca 2p X-ray photoelectron spectroscopy profiles; and **d** field-emission scanning electron microscopy images of **A** bare Ga_2_O_3_; Ga_2_O_3__Ca_*x* with a Ca/Ga molar ratio *x* of **B** 0.32 mol%, **C** 0.62 mol%, **D** 1.1 mol%, **E** 2.1 mol%, and **F** 3.3 mol%; **G** CaGa_4_O_7_ (in **a**, **b**), and **H** CaO (in **c**).

The actual amounts of the Ca species loaded into Ga_2_O_3_ at different CaCl_2_ concentrations were measured using inductively coupled plasma optical emission spectrometry (ICP-OES) (Supplementary Table 2). We found that almost all the Ca species were loaded into the Ga_2_O_3_ photocatalyst when the CaCl_2_ concentration was <0.001 mol L^−1^. However, not all the Ca species could be loaded into Ga_2_O_3_ at higher CaCl_2_ concentrations. Note that even when no CaCl_2_ was added during the preparation of Ga_2_O_3_, trace amounts of Ca were detected in Ga_2_O_3_, which is likely due to Ca impurities present in the experimental vessels or precursor reagents. Hereinafter, we refer to the Ca-loaded Ga_2_O_3_ photocatalysts as Ga_2_O_3__Ca_*x* (*x* = 0.32, 0.62, 1.1, 1.6, 2.1, 3.3 mol%) based on the Ca/Ga molar ratio determined by ICP-OES. Figure 1a shows the X-ray diffraction (XRD) patterns of the bare Ga_2_O_3_, Ga_2_O_3__Ca_*x*, and CaGa_4_O_7_ photocatalysts. As indicated, gradual changes in the diffraction peaks assigned to the (020), (311), (400), (002), and (330) facets of CaGa_4_O_7_ (JSPDS 01-071-1613) were observed as the amount of Ca species was increased. In general, a high Ca loading is favorable for the formation of CaGa_4_O_7_. We observed no distinct shifts in the diffraction peaks for the Ga_2_O_3__Ca_*x* samples compared with those of bare Ga_2_O_3_. As the ionic radius of Ca^2+^ (0.099 nm)^32^ is larger than that of Ga^3+^ (0.062 nm)^33^, the unshifted XRD peaks imply that Ca^2+^ does not act as a dopant in the bulk Ga_2_O_3_ lattice. However, there was a clear increase in the peak intensity at 2*θ* = 30.1° and an apparent decrease in that at 2*θ* = 30.5° with increasing amount of Ca species (Fig. 1b), which are possibly ascribed to the formation of CaGa_4_O_7_ species on Ga_2_O_3_. The increased intensity of the Ca 2p X-ray photoelectron spectroscopy (XPS) peak (Fig. 1c) also indicates that the amount of Ca species on the Ga_2_O_3_ surface increased with increasing Ca levels. In addition, the XPS peak locations in the Ca 2p spectra of the Ga_2_O_3__Ca_*x* photocatalysts are similar to those of CaGa_4_O_7_, but different from those of CaO. The Ca 2p XPS profiles suggest that a thin CaGa_4_O_7_ layer forms on the Ga_2_O_3_ surface and that the amount of CaGa_4_O_7_ increases as the amount of Ca is increased. We further confirmed the morphological changes in the Ga_2_O_3__Ca sample by field-emission scanning electron microscopy (SEM), as shown in Fig. 1d. Both ends of the Ga_2_O_3_ nanoparticles gradually sharpened and their surfaces became smoother as the amount of Ca species increased, especially when the Ca amount was higher than 1.1 mol%. This smoothing of the Ga_2_O_3_ surfaces with increasing Ca/Ga molar ratio resulted in a decrease in the Brunauer–Emmett–Teller (BET) specific surface area of Ga_2_O_3__Ca_*x* (Supplementary Fig. 4), which is attributable to the modification of CaGa_4_O_7_, as we confirmed from the XRD patterns and the XPS results that a CaGa_4_O_7_ layer was formed on the Ga_2_O_3_ surface.

**Fig. 2:**
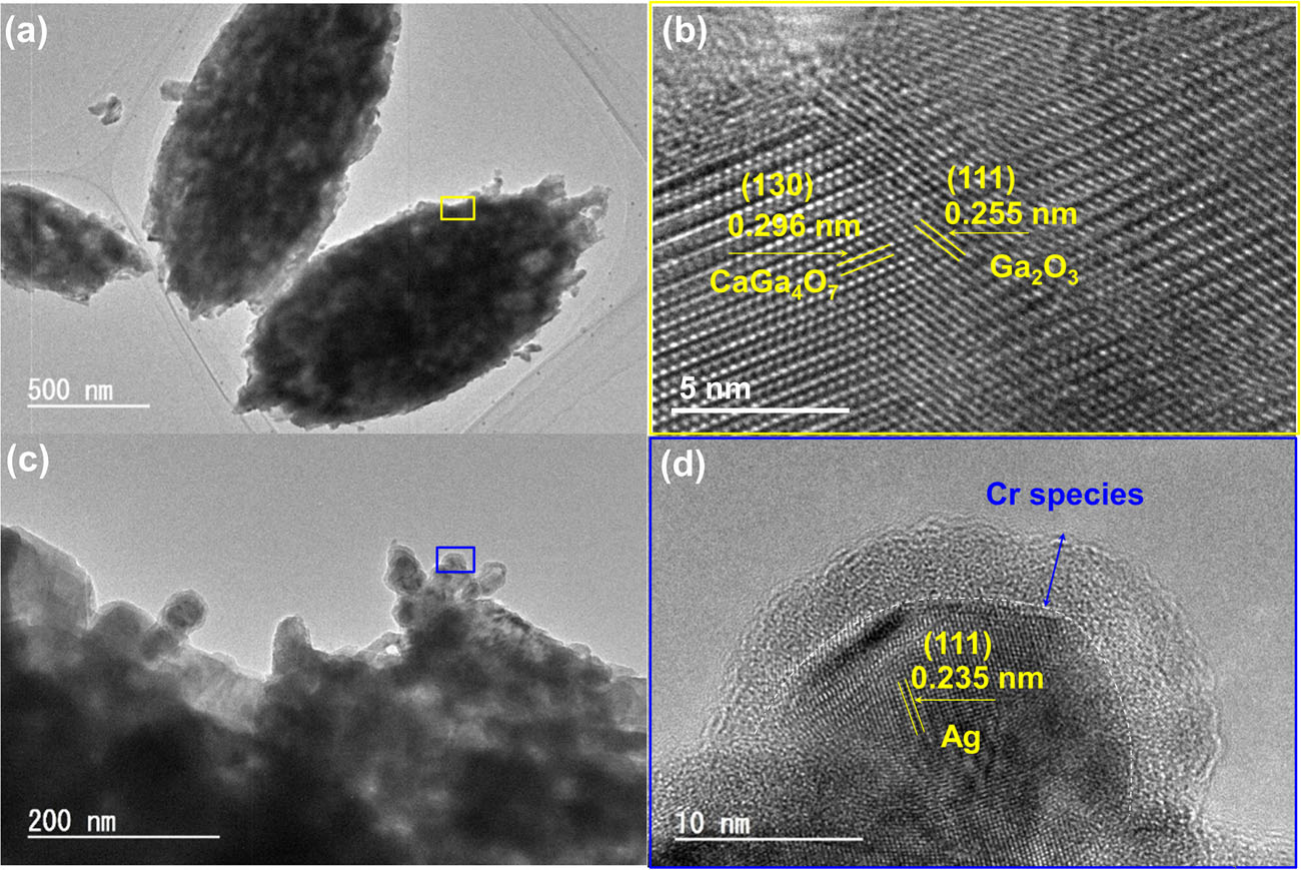
Transmission electron microscopy (TEM) images of the photocatalysts. TEM images of **a** Ga_2_O_3__Ca and **c** Ag@Cr/Ga_2_O_3__Ca. High-resolution TEM images of **b** Ga_2_O_3__Ca and **d** Ag@Cr/Ga_2_O_3__Ca. Note that **b**, **d** are enlarged TEM images of the marked areas in **a**, **c** indicated by yellow and blue boxes, respectively.

The close linkage between CaGa_4_O_7_ and Ga_2_O_3_ on the Ga_2_O_3_ surface was confirmed by field-emission transmission electron microscopy (TEM) and high-resolution TEM (HRTEM) (Fig. 2). The marked lattice spacings (0.296 and 0.255 nm) in Fig. 2b correspond to the (130) and (111) planes of CaGa_4_O_7_ and Ga_2_O_3_, respectively. The core–shell-structured Ag@Cr cocatalyst was successfully loaded onto the Ga_2_O_3__Ca surface using the photodeposition method (Fig. 2c, d), as reported previously by us^31^.

### Role of the Ca species

**Fig. 3:**
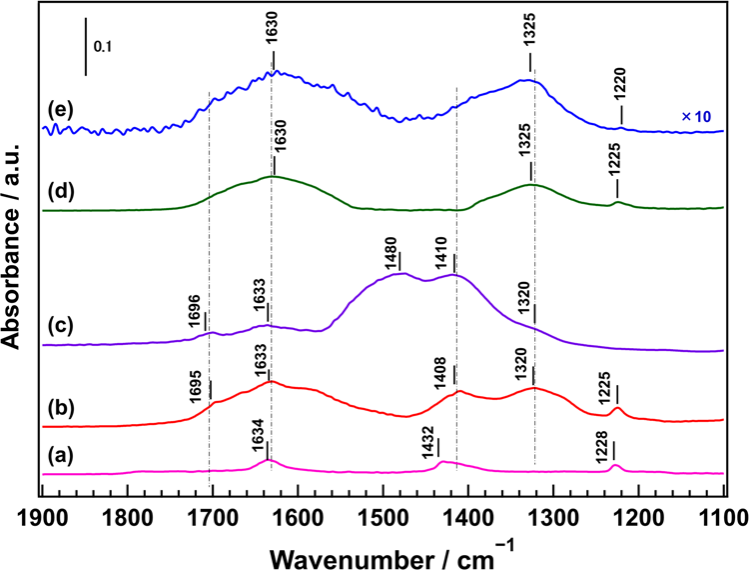
Fourier transform infrared spectra of CO_2_ adsorption. CO_2_ adsorbed on: **a** Ga_2_O_3_, **b** Ga_2_O_3__Ca_1.1, **c** CaO, **d** Ga_2_O_3__Ca_3.3, and **e** CaGa_4_O_7_ after introducing ~0.2 Torr of CO_2_.

Figure 3 shows the Fourier transform infrared (FTIR) spectra of the CO_2_-adsorbed samples after introducing CO_2_ at ~0.2 Torr. When CO_2_ was introduced into the Ga_2_O_3_ sample, three absorbance peaks were observed at 1634, 1432, and 1225 cm^–1^, which can be ascribed to asymmetric CO_3_ stretching vibrations [*ν*_as_(CO_3_)], symmetric CO_3_ stretching vibrations [*ν*_s_(CO_3_)] of monodentate bicarbonate species (m-HCO_3_-Ga), and OH deformation vibrations [*δ*(OH)], respectively^34–36^. The absorbance peaks at 1699 and 1636 cm^–1^ for the CO_2_-adsorbed CaO sample can be attributed to bridging carbonate stretching and asymmetric CO_3_ stretching vibrations [*ν*_as_(CO_3_)] of the bicarbonate species, respectively. The broad structureless absorbance peaks between 1480 and 1318 cm^–1^ can be attributed to the symmetric and asymmetric CO_3_ stretching of unidentate carbonate, as well as the symmetric CO_3_ stretching [*ν*_s_(CO_3_)] of bicarbonate^37–41^. When the Ga_2_O_3_ surface was modified with a small amount of Ca species, absorbance peaks attributable to CO_2_ adsorption by both Ga_2_O_3_ and CaO were observed after CO_2_ was introduced into the Ga_2_O_3__Ca_1.1 sample. However, when the Ga_2_O_3_ surface was modified with large amounts of Ca species, the absorbance peaks attributed to CO_2_ adsorption on Ga_2_O_3_ had low intensity and mainly corresponded to the broad peaks derived from the adsorption of CO_2_ on CaGa_4_O_7_. Supplementary Fig. 5 shows the FTIR spectra of CO_2_-adsorbed Ga_2_O_3_, Ga_2_O_3__Ca_1.1, Ga_2_O_3__Ca_3.3, and CaGa_4_O_7_ samples after introducing the same amount of CO_2_ at various pressures in the 0.1–40.0 Torr range. CO_2_ was adsorbed significantly more on the Ga_2_O_3__Ca_1.1 surface than on the Ga_2_O_3_ surface due to its adsorption at both Ga and Ca sites. However, the CaGa_4_O_7_ surface was not conducive to CO_2_ adsorption; therefore, CO_2_ adsorbed less onto the Ga_2_O_3__Ca_3.3 surface than the Ga_2_O_3__Ca_1.1 surface.

**Fig. 4:**
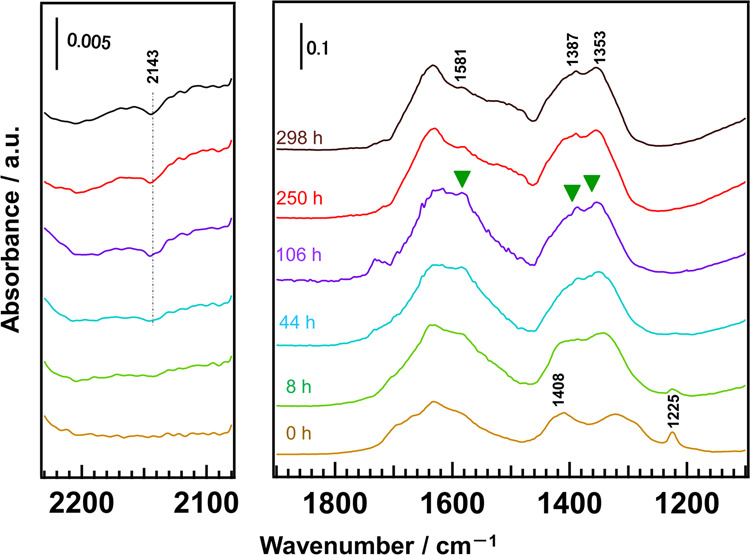
FTIR spectra of CO_2_ adsorption under photoirradiation. Difference FTIR spectra of the adsorbed CO_2_ species on Ga_2_O_3__Ca_1.1 under photoirradiation for different hours. ~2.0 Torr of CO_2_ was introduced into the instrument.

Figure 4 shows the FTIR spectra of the adsorbed CO_2_ species on Ga_2_O_3__Ca_1.1 after different durations of photoirradiation. As the photoirradiation time increased from 0 to 106 h, the bands at 1225 [*δ*(OH)-Ga] and 1408 cm^–1^ [*ν*_s_(CO_3_)-Ca] decreased and vanished after 104 h. At the same time, new bands gradually appeared at 1581, 1388, and 1353 cm^–1^ (asymmetric CO_2_ stretching [*ν*_as_(CO_2_)], CH deformation [*δ*(CH)], and symmetric CO_2_ stretching [*ν*_s_(CO_2_)] assigned to formate species (HCOO–Ga/Ca), respectively)^34–36^. As the photoirradiation continued, the formate species were consumed and gaseous CO (fundamental vibration band at 2143 cm^−1^)^42^ was formed simultaneously. This result indicates that the bicarbonate species is the intermediate during the photocatalytic conversion of CO_2_, and the formates transform into CO with photoirradiation, which is consistent with our previous results^43,44^. It is worth mentioning that in addition to the presence of intermediate species on the Ga_2_O_3_ surface ([*δ*(OH)–Ga]), the modification by Ca species further increased the amount of intermediate on Ga_2_O_3__Ca_1.1. As the photocatalytic conversion of H^+^ into H_2_ and the conversion of CO_2_ into CO are two competing processes in an aqueous solution, the high adsorption of CO_2_ at the base site leads to high photocatalytic activity and selectivity toward CO evolution during the photocatalytic conversion of CO_2_ by H_2_O.

**Fig. 5:**
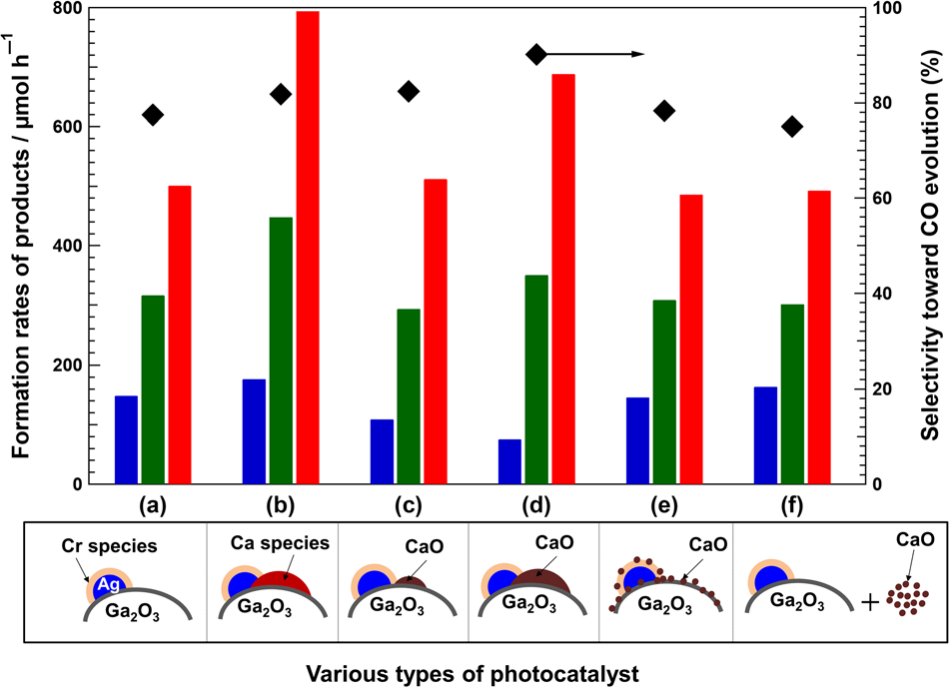
Product formation rates and selectivity. Rates of formation of H_2_ (blue bars), O_2_ (green bars), and CO (red bars), as well as selectivity toward CO evolution (black diamonds) for various photocatalysts: **a** Ag@Cr/Ga_2_O_3_; **b** Ag@Cr/Ga_2_O_3__Ca_1.1; **c** Ag@Cr/(1.1 mol%CaO/Ga_2_O_3_), namely Ga_2_O_3_ physically mixed with 1.1 mol% of CaO by grinding before loading the Ag@Cr cocatalyst; **d** Ag@Cr/(30 mol%CaO/Ga_2_O_3_), which is similar to **c** except for using 30 mol% of CaO; **e** Ag@Cr/Ga_2_O_3_ + 30 mol%CaO, namely Ag@Cr/Ga_2_O_3_ physically mixed with 30 mol% of CaO by grinding; and **f** Ag@Cr/Ga_2_O_3_ and 30 mol% of CaO without mixing before adding into the reaction solution. Schematic structures of the photocatalysts are shown at bottom. Photocatalyst powder: 0.5 g, reaction solution volume: 1.0 L, additive: 0.1 M NaHCO_3_, CO_2_ flow rate: 30 mL min^−1^, light source: 400 W high-pressure Hg lamp.

In order to demonstrate that the presence of CaO on the Ga_2_O_3_ surface enhances the photocatalytic activity and selectivity during the photocatalytic conversion of CO_2_ into CO, we investigated the photocatalytic performance during the conversion of CO_2_ by H_2_O over various Ag@Cr/CaO/Ga_2_O_3_ photocatalysts, the results of which are shown in Fig. 5. We found that the Ag@Cr/Ga_2_O_3__Ca_1.1 photocatalyst (with a low amount of CaO generated on the Ga_2_O_3_ surface) significantly enhanced the rate of CO formation during the photocatalytic conversion of CO_2_ by H_2_O compared with bare Ag@Cr/Ga_2_O_3_ (Fig. 5a, b). However, no significant change in the rate of CO formation and selectivity toward CO evolution was observed for the sample labeled “Ag@Cr/(1.1 mol%CaO/Ga_2_O_3_)” (in which 1.1 mol% CaO was physically loaded onto Ga_2_O_3_ by grinding before loading Ag@Cr cocatalyst onto the CaO/Ga_2_O_3_ surface) as compared to bare Ga_2_O_3_ (Fig. 5c). Because uncalcined CaO-loaded Ga_2_O_3_ easily dissolves in H_2_O, we increased the CaO loading on the Ga_2_O_3_ surface to 30 mol% using the same grinding method (labeled “Ag@Cr/(30 mol%CaO/Ga_2_O_3_)”), which resulted in an increased rate of CO formation and a decrease in H_2_ formation (Fig. 5d). However, no improvement in photocatalytic activity and selectivity was observed when 30 mol% CaO was mixed with the prepared Ag@Cr/Ga_2_O_3_ and ground together (Fig. 5e) or when they were directly mixed in the reaction solution (Fig. 5f). These results clearly reveal that the addition of CaO on the Ga_2_O_3_ surface enhances the rate of CO formation and suppresses that of H_2_ during the photocatalytic conversion of CO_2_ by H_2_O. In addition, the tight junction between Ga_2_O_3_, CaO, and the Ag@Cr cocatalyst is crucial for the superior photocatalytic activity and selectivity of the photocatalyst for the conversion of CO_2_ into CO. In our previous work, we confirmed that Ag acts as an active site while the Cr(OH)_3_·H_2_O layer exterior to the Ag core increases CO_2_ adsorption^30,31^. Hence, the Ag@Cr cocatalyst should be loaded at the CaO/Ga_2_O_3_ interface in order to facilitate contact between the CaO-adsorbed CO_2_ species and the Ag active sites.

Notably, although CaGa_4_O_7_ exhibited high selectivity toward H_2_ evolution, the H_2_ formation rate for CaGa_4_O_7_ was significantly lower than that for Ga_2_O_3__Ca_3.3 (for the product formation rates over Ag-Cr/Ga_2_O_3__Ca_3.3 see Supplementary Fig. 6). This indicates that the presence of CaGa_4_O_7_ on the Ga_2_O_3_ surface enhances the overall photocatalytic efficiency during the photocatalytic reaction, including CO_2_ conversion and water splitting. The Mott–Schottky plot (Supplementary Fig. 7) and the absorption spectra converted from the diffuse reflectance spectra using the Kubelka–Munk equation (Supplementary Fig. 8) enabled us to estimate the conduction band (CB) and valence band (VB) positions of Ga_2_O_3_, Ga_2_O_3__Ca_0.62, and CaGa_2_O_7_, as shown in Supplementary Fig. 9. Since the CB and VB of Ga_2_O_3_ are both more positive than those of CaGa_4_O_7_, a heterojunction system between Ga_2_O_3_ and CaGa_4_O_7_ can be formed that greatly improves the spatial separation efficiency of the photogenerated carriers^45^. Therefore, CaGa_4_O_7_/Ga_2_O_3_ exhibited a much higher photocatalytic efficiency than bare Ga_2_O_3_ and CaGa_4_O_7_.

**Fig. 6:**
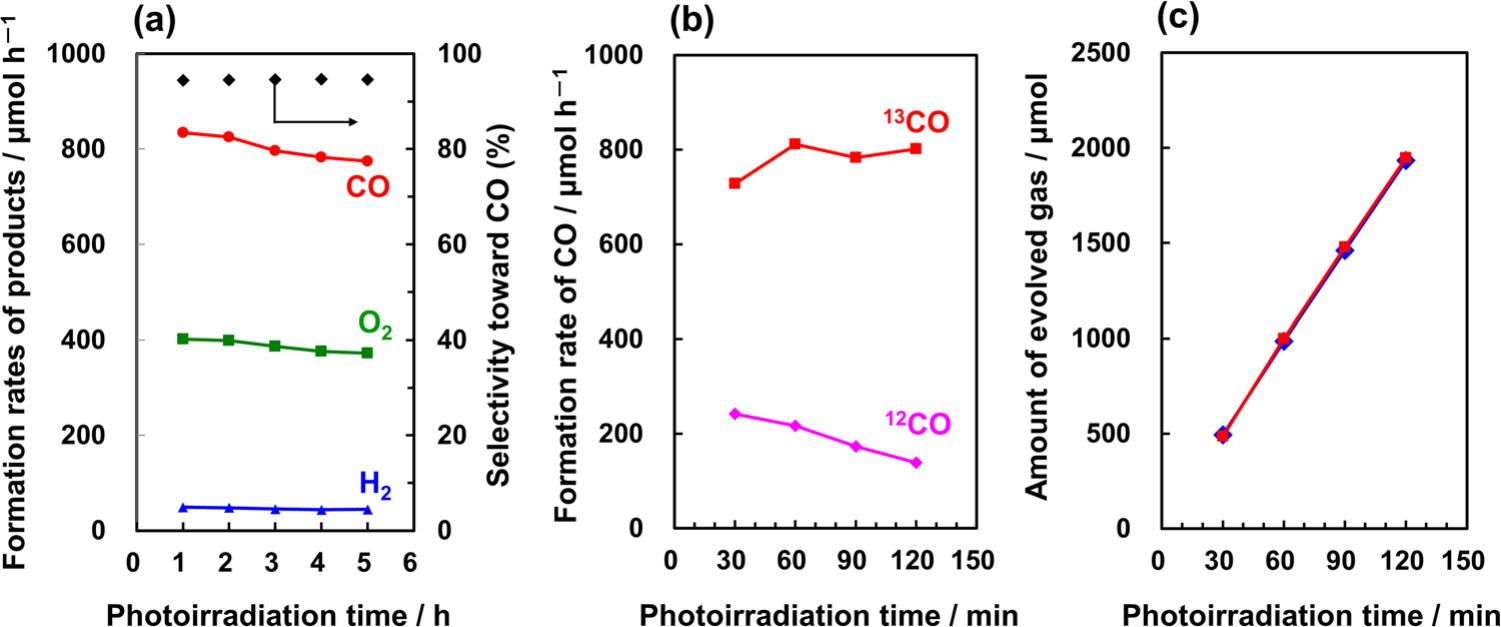
CO, O_2_, and H_2_ formation data. **a** Formation rates of H_2_ (blue triangles), O_2_ (green squares), and CO (red circles), and selectivity toward CO evolution (black diamonds) for the photocatalytic conversion of CO_2_ by H_2_O; **b** ^12^CO and ^13^CO detected by MS (*m*/*z* = 28 and 29) from the photocatalytic conversion of ^13^CO_2_ by H_2_O; **c** CO time-course as determined by MS (red squares) and GC (blue diamonds) for the photocatalytic conversion of ^13^CO_2_ by H_2_O.

We expect that by exploiting the high CO_2_ adsorption of CaO and the high photocatalytic efficiency of CaGa_4_O_7_/Ga_2_O_3_, we can further improve the photocatalytic activity and selectivity of the photocatalyst to maximize the conversion of CO_2_ into CO by H_2_O. Figure 6a shows the formation rates of H_2_, O_2_, and CO during the photocatalytic conversion of CO_2_ by H_2_O for the Ga_2_O_3__Ca_3.3 photocatalyst physically mixed with 30 mol% of CaO and Ag@Cr as the cocatalyst. As indicated, a high formation rate of CO (>835 µmol h^–1^) was achieved, in addition to an excellent selectivity toward CO evolution (>95%), with a stoichiometric amount of evolved O_2_. Both ^12^CO and ^13^CO were detected using quadrupole mass spectrometry (MS), and the peaks at *m*/*z* = 28 and *m*/*z* = 29 were located at the same positions as those detected by gas chromatography (GC) during the photocatalytic conversion of ^13^CO_2_ (for the isotopic lead experiments see Supplementary Fig. 10). Indeed, our results indicate that the detected ^12^CO was produced from the reduction of ^12^CO_2_ derived from the NaHCO_3_ additive in the solution^43^. As shown in Fig. 6b, with the consumption of ^12^CO_2_ derived from NaHCO_3_, the amount of generated ^12^CO gradually decreased, while the ^13^CO content increased under continuous bubbling of ^13^CO_2_. The total amounts of ^13^CO and ^12^CO detected by MS were consistent with the amount of CO detected by GC (Fig. 6c), which indicates that the CO was generated as the reduction product of either CO_2_ introduced in the gas phase or from NaHCO_3_, rather than from any organic contaminants on the photocatalyst surface. The converted concentration of CO based on the CO formation rate was found to be 11,531 ppm, indicating that ~1.2% of CO_2_ in the gas phase was transformed into CO (see Supplementary Information for the calculation details. The actual amounts of CO detected are shown in Supplementary Movie 1).

**Fig. 7:**
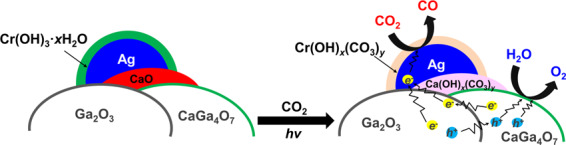
A plausible reaction mechanism. Schematic illustration of the mechanism for the photocatalytic conversion of CO_2_ into CO over Ag@Cr/CaO/CaGa_4_O_7_/Ga_2_O_3_.

In our previous work, we had found that basic oxides and hydroxides such as Cr(OH)_3_^31^, SrO^44^, and rare earth (RE) hydrates and oxides^28^ function as good CO_2_ storage materials by generating the corresponding (hydroxy)carbonate compounds (e.g., Cr(OH)_x_(CO_3_)_y_ and RE_2_(OH)_2(3−x)_(CO_3_)_x_), and they improve the photocatalytic activity and selectivity toward CO evolution. Now, we propose a possible mechanism for the photocatalytic conversion of CO_2_ by H_2_O over Ag@Cr/CaO/CaGa_2_O_7_/Ga_2_O_3_, as shown in Fig. 7. During the photocatalytic conversion of CO_2_ in an aqueous solution of NaHCO_3_, the Cr(OH)_3_·H_2_O and CaO species that are in close contact with Ag particles easily form (hydroxy)carbonate species (named M(OH)_x_(CO_3_)_y_, M=Cr or Ca)^31^, which greatly increase the concentration of CO_2_-related species around the Ag active sites, thereby improving selectivity for the photocatalytic conversion of CO_2_ into CO instead of water splitting. On the other hand, the Ga_2_O_3_/CaGa_4_O_7_ heterojunction improves the efficiency for spatial separation of the photogenerated carriers, which also increases the photocatalytic activity for the conversion of CO_2_ into CO. Moreover, while the Cr(OH)_3_·xH_2_O shell outside the Ag particle can be oxidized to Cr^6+^ and dissolve into the solution during the photocatalytic conversion of CO_2_^46^, the presence of CaO around the Ag active site compensates for the reduced activity from the dissolution of Cr species. As a result, Ag@Cr/Ga_2_O_3__Ca is photocatalytically much more stable than Ag@Cr/Ga_2_O_3_.

Herein, we reported the photocatalytic conversion of CO_2_ using a Ag@Cr/CaO/CaGa_4_O_7_/Ga_2_O_3_ photocatalyst, in which a satisfactory CO formation rate (>835 µmol h^−1^) and an excellent selectivity toward CO evolution (95%) were achieved with the stoichiometric production of O_2_ as the oxidation product of H_2_O. Through the use of various characterization techniques, we found that the CaO and CaGa_4_O_7_ formed on the Ga_2_O_3_ surface improved the adsorption of CO_2_ at basic sites in addition to enhancing the total photocatalytic efficiency. In addition, the physical mixing of CaGa_4_O_7_/Ga_2_O_3_ with CaO was a particularly simple and convenient technique for exploiting the high CO_2_ adsorption ability of CaO and the high photocatalytic efficiency of CaGa_4_O_7_/Ga_2_O_3_. These results are of particular interest, considering that previously, only insufficient amounts of CO_2_ reduction products were produced during artificial photosynthesis.

## Methods

Ca-modified Ga_2_O_3_ (Ga_2_O_3__Ca) was prepared using the ammonia precipitation method reported by Sakata et al.^47^. In this method, Ga(NO_3_)_3_∙*n*H_2_O (12.6 g) was dissolved in 200 mL of deionized water or CaCl_2_ solution in ultrapure water at various concentrations. Hydroxylation was carried out by dripping an ammonium hydroxide solution until the pH level reached 9.1. The obtained hydroxides were centrifuged and dried overnight. The Ga_2_O_3__Ca sample was obtained by calcining the precursor at 1273 K for 10 h. Ag@Cr/Ga_2_O_3__Ca was synthesized using the photodeposition method reported in our previous work^30^. In this method, the as-prepared Ga_2_O_3__Ca powder (1.0 g) was dispersed in ultrapure water (1.0 L) containing the necessary amounts of silver nitrate (AgNO_3_) and chromium (III) nitrate (Cr(NO_3_)_3_). The suspension was purged with Ar gas and irradiated under a 400 W high-pressure Hg lamp with Ar gas flowing for 1.0 h, followed by filtration and drying at room temperature (~298 K). The Ag/Ga and Cr/Ga molar ratios were both 1.0 mol%.

### Characterization

The as-prepared Ga_2_O_3__Ca samples were characterized using the following techniques: XRD (Model: Multiflex, Rigaku Corporation, Japan) with Cu Kα radiation (*λ* = 0.154 nm); XPS (Model: ESCA 3400, Shimadzu Corporation, Japan) with Mg Kα radiation; SEM (Model: SU-8220, Hitachi High-Technologies Corporation, Japan); TEM (Model: JEM-2100F, JEOL Ltd, Japan); and UV–Visible spectroscopy (V-650, JASCO) with an integrated sphere accessory. The BET surface areas of the photocatalyst samples were determined from their N_2_-adsorption isotherms at 77 K using a volumetric gas-adsorption measuring instrument (Model: BELSORP-miniII, MicrotracBEL Corp. (formerly BEL Japan, Inc.), Japan). Prior to these measurements, each sample was evacuated at 473 K for 1 h using a sample pretreatment system (Model: BELPREP-vacII, MicrotracBEL Corp. (formerly BEL Japan, Inc.), Japan). ICP-OES (Model: iCAP7400, Thermo Fisher Scientific, USA) was used to determine the actual amounts of Ca modified on the Ga_2_O_3_ surface. The FTIR spectra of the adsorbed carbon species were recorded using an FTIR spectrometer (Model: FT/IR-4700, JASCO International Co., Ltd., Japan) equipped with a mercury–cadmium–tellurium (MCT) detector and cooled with liquid N_2_ in the transmission mode at 303 K. Each sample (~30 mg) was pressed into a wafer (diameter: 10 mm) and introduced into the instrument in a cylindrical glass cell with calcium fluoride (CaF_2_) windows. The wafer was evacuated at 673 K for 30 min before being examined, followed by treatment with O_2_ at ~40 Torr for 30 min, after which the wafer was evacuated for 30 min and cooled to 303 K. The data for each FTIR spectrum were obtained from 128 scans with a resolution of 4 cm^−1^. The energy gap of the band structure and flat band potential of the Ga_2_O_3__Ca samples were determined using the Davis–Mott and Mott–Schottky equations, respectively; the experimental details are provided in the Supplementary Information.

### Photocatalytic reaction

The photocatalytic reduction of CO_2_ was carried out using a flow system with an inner irradiation-type reaction vessel. The synthesized photocatalyst (0.5 g) was dispersed in ultrapure water (1.0 L) containing 0.1 M sodium bicarbonate (NaHCO_3_). The CO_2_ was bubbled into the solution at a flow rate of 30 mL min^−1^. The suspension was illuminated using a 400 W high-pressure Hg lamp with a quartz filter, and the assembly was connected to a water-cooling system. The amounts of evolved H_2_ and O_2_ were detected using a gas chromatography system fitted with a thermal conductivity detector (TCD-GC, Model: GC-8A, Shimadzu Corporation, Japan) and a 5A molecular sieve (MS 5A) column, and Ar was used as the carrier gas. The amount of evolved CO was analyzed using a gas chromatography system fitted with a flame ionization detector (FID-GC, Model: GC-8A, Shimadzu Corporation, Japan), a methanizer, and a ShinCarbon ST column, and N_2_ was used as the carrier gas. High-performance liquid chromatography (Model: LC-4000, JASCO, USA) was used to detect the presence of liquid products.

In the isotope experiment, ^12^CO_2_ was replaced by ^13^CO_2_. The formation rates of H_2_, O_2_, ^12^CO, and ^13^CO under photoirradiation were detected using a quadrupole mass spectrometer (BELMASS, Microtrac BEL) combined with a TCD-GC detector.

## Supplementary information

Supplementary Information

Description of Additional Supplementary Files

Supplementary Movie 1

Peer Review File

## Data Availability

The datasets generated during and/or analysed during the current study are available in the [figshare] repository, [https://figshare.com/s/84a5d675a273e507fb55 and/or 10.6084/m9.figshare.12927422].
