# Supplementary material for: Enhanced CO evolution for photocatalytic conversion of CO2 by H2O over Ca modified Ga2O3
Source: Commun Chem. 2020 Oct 9;3:137. doi: 10.1038/s42004-020-00381-2 (PMC9814714; doi:10.1038/s42004-020-00381-2)
Supplement: Supplementary file 1 — Supplementary Information [file 42004_2020_381_MOESM1_ESM.pdf]

Supplementary Information

Enhanced CO Evolution for Photocatalytic Conversion of CO_2 by H_2O over Ca Modified Ga_2O_3

Rui Pang[†], Kentaro Teramura[†,‡*], Masashige Morishita[†], Hiroyuki Asakura[†,‡], Saburo Hosokawa[†,‡], Tsunehiro Tanaka[†,‡*]

[†]Department of Molecular Engineering, Graduate School of Engineering, Kyoto University, Kyotodaigaku Katsura, Nishikyo-ku, Kyoto 615-8510, Japan

[‡]Element Strategy Initiative for Catalysts & Batteries (ESICB), Kyoto University, 1-30 Goryo-Ohara, Nishikyo-ku, Kyoto 615-8245, Japan

E-mail address: teramura@moleng.kyoto-u.ac.jp; tanakat@moleng.kyoto-u.ac.jp

Supplementary Methods

Preparation of photocatalysts electrodes

Ga_2O_3 and Ga_2O_3_Ca photocatalysts electrodes were prepared on a fluorine doped tin oxide (FTO) glass via an electrophoresis deposition method. 100 mg of photocatalyst powder was added into 50 mL of an acetone solution containing 10 mg of iodine as an electrolyte, and then the photocatalyst powder was dispersed thoroughly by an ultrasonication. Prior to use, FTO glass (AGC fabritech Co., Ltd) was washed with aceton and 2-propanol solution in turn. Two FTO glasses were immersed in the solution with facing each other, and direct current (DC) was applied between the two FTO glasses by using an electrochemical measurement system (HZ-5000, Hokuto Denko Corp.), at 0.1 mA stable current (2 min) for measurements in an aqueous solution, and at 10.0 V stable voltage (5 min) for those in an organic solution. After drying at room temperature in air, prepared photocatalyst electrode was heated at 473 K for 2 h in order to remove the residual iodine.

Electrochemical impedance measurements

Electrochemical impedance measurements were performed using a three-electrode electrochemical cell consisting of the prepared photocatalyst/FTO electrode, Ag/AgCl electrode, and Pt wire as working electrode, reference electrode, and counter electrode, respectively. Prior to the measurements, the dissolved air in the electrolyte solution was completely removed by N_2 gas flow. An aqueous Na_2SO_4 solution (0.1 M) was used as an electrolyte solution. The imaginary component of the impedance (Z”) of the equivalent circuit including photocatalyst/FTO electrode was evaluated at an alternating current frequency of 39.8, 31.6, and 25.1 kHz with a sweeping applied voltage from 0.5 to −0.5 V vs. Ag/AgCl by an electrochemical measurement system (HZ-5000, Hokuto Denko

Corp.). The capacitance (***C***) of the circuit was calculated from the imaginary component of the impedance (***Z***") using the relationship,

$$|Z''| = 1/(2\pi f C) \qquad \text{Eq. S1}$$

where π and f means the circumference ratio and the frequency of the alternating current. The value of the flat band potential (hereinafter "E_{FB}") for the working electrode was estimated by using the resulted value of ***C*** in accordance with Mott-Schottky equation,

$$C^{-2} = (2/\varepsilon\varepsilon_0 A^2 e N_D)\,(E - E_{FB} - k_B T/e) \qquad \text{Eq. S2}$$

where: ***C*** and ***A*** are the interfacial capacitance and area, respectively, N_D the number of donors, ***E*** the applied potential, k_B Boltzmann's constant, ***T*** the absolute temperature, ε the dielectric constant of the semiconductor, ε_0 the permittivity of free space, and ***e*** is the electronic charge. Therefore, the value of E_{FB} should be obtained from the intercept on *x*-axis in the plot of C^{-2} versus the applied potential ***E***.

The conversion rate of CO_2 into CO:

Flowing rate of CO_2 = 30 mL min^{-1}

Formation rate of CO = 835 μmol h^{-1}

The concentration of CO in 30 mL min^{-1} of CO_2 = V_{co}/V_{CO2} = (835 × 10^{-6} × 8.31 × 10^{3} × 303/ (1.013 × 10^{5}))/(30 × 60 × 10^{-3}) × 10^{6}= 11531 ppm

The conversion rate of CO_2 to CO = 1.15% ≈ 1.2%

Supplementary Table 1. Summary of photocatalysts for the conversion of CO_2 into CO using H_2O as an electron donor under similar experimental conditions.

Catalyst	Weight /g	Light source	Co-catalyst	Additive	Activity / μmol h^{-1}			Selec. to CO/%	Ref.
					H_2	O_2	CO		
$BaLa_4Ti_4O_{15}$	0.3	400-W Hg lamp	2.0 wt% Ag	None	10.0	16.0	22.0	68.8	1
$NaTaO_3$:Ba	1.0	400-W Hg lamp	3.0 wt% Ag	0.1 M $NaHCO_3$	31.0	170[a]	318	91.0	2
$CaTiO_3$	0.3	100 W Hg lamp	3.5 wt% Ag	1.0 M $NaHCO_3$	3.10	25.0	54.0	94.0	3
$Na_2Ti_6O_{13}$	0.2	100 W Hg lamp	1.0 wt% Ag	0.5 M $NaHCO_3$	1.60	0.70[a]	4.60	74.0	4
$La_2Ti_2O_7$	1.0	400-W Hg lamp	1.0 wt% Ag	0.1 M $NaHCO_3$	4.09	5.30	5.20	51.5	5
$ZnGa_2O_4$	1.0	400-W Hg lamp	1.0 wt% Ag	0.1 M $NaHCO_3$	8.50	74.3	155	95.0	6
$ZnGa_2O_4$/ Ga_2O_3	1.0	400-W Hg lamp	1.0 wt% Ag	0.1 M $NaHCO_3$	16.9	70.1	117	87.4	7
SrO/Ta_2O_5	1.0	400-W Hg lamp	3.0 wt% Ag	0.1 M $NaHCO_3$	3.80	5.10	6.80	64.2	8
$KCaSrTa_5O_{15}$	0.5	400-W Hg lamp	0.5 wt% Ag	0.1 M $NaHCO_3$	15.0	46.0	97.0	86.7	9
$ZnTa_2O_6$	1.0	400-W Hg lamp	1.0 wt% Ag	0.1 M $NaHCO_3$	25.1	18.6	19.3	43.4	10
$Sr_2KTa_5O_{15}$	1.0	400-W Hg lamp	1.0 wt% Ag	0.1 M $NaHCO_3$	8.30	34.3	65.5	88.8	11
$K_2YTa_5O_{15}$	1.0	400-W Hg lamp	1.0 wt% Ag	0.1 M $NaHCO_3$	16.2	43.2	91.9	85.0	12
$Sr_{1.6}K_{0.37}Na_{1.43}Ta_5O_{15}$	1.0	400-W Hg lamp	1.0 wt% Ag	0.1 M $NaHCO_3$	16.0	53.7	94.6	85.5	13
$SrNb_2O_6$	0.5	400-W Hg lamp	0.5 wt% Ag	0.1 M $NaHCO_3$	1.10	24.8	51.2	97.9	14
Mg-Al LDH/Ga_2O_3	1.0	400-W Hg lamp	1.0 wt% Ag	0.1 M $NaHCO_3$	131	167	212	61.7	15
Pr/Ga_2O_3	0.5	400-W Hg lamp	1.0 wt% Ag	0.1 M $NaHCO_3$	64.7	150	249	79.4	16
Yb-Zn/Ga_2O_3	0.5	400-W Hg lamp	1.0 wt% Ag	0.1 M $NaHCO_3$	37.6	103	150	80.0	17
Ga_2O_3	0.5	400-W Hg lamp	1.0 mol% (Ag-Cr)	0.1 M $NaHCO_3$	92.9	281	480	83.8	18
Ga_2O_3_Ca	0.5	400-W Hg lamp	1.0 mol% (Ag-Cr)	0.1 M $NaHCO_3$	49.0	402	835	94.5	This work

[a] Estimated from the figure mentioned in the paper.

Supplementary Table 2. Comparison between the calculated Ca/Ga molar ratios and those measured by ICP-OES at different $CaCl_2$ concentrations.

$CaCl_2$ concentration (mol L^{-1})	Ca/Ga molar ratio (mol%) (Calculated)	Ca/Ga molar ratio (mol%) (ICP-OES)
0.0000	0.00	0.056
0.0005	0.31	0.32
0.0010	0.63	0.62
0.0020	1.3	1.1
0.0030	2.0	1.6
0.0050	3.3	2.1
0.0100	6.5	3.3

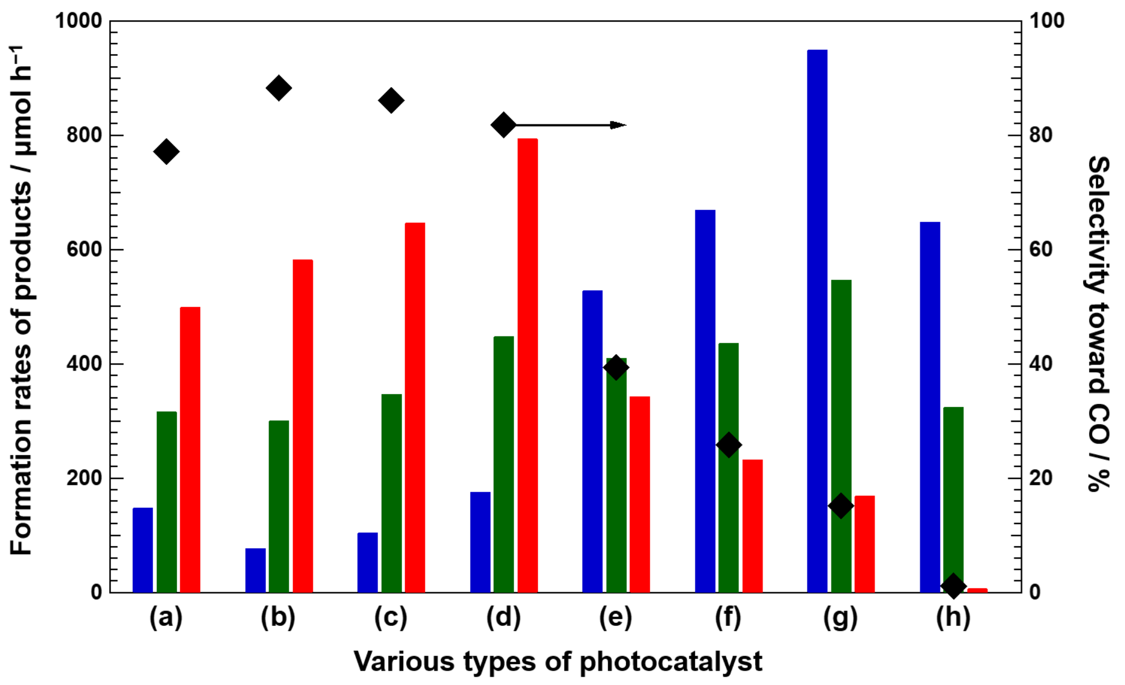

Supplementary Figure 1. Product formation rates and selectivity. Formation rates of H_2 (blue bars), O_2 (green bars), and CO (red bars) and selectivity toward CO evolution (black diamonds) for the (a) Ag-Cr/Ga_2O_3, Ag-Cr/Ga_2O_3_Ca_*x* with a Ca/Ga molar ratio *x* of (b) 0.32 mol%, (c) 0.62 mol%, (d) 1.1 mol%, (e) 1.6 mol%, (f) 2.1 mol%, and (g) 3.3 mol%, and (h) Ag-Cr/$CaGa_4O_7$ during the photocatalytic conversion of CO_2 by H_2O. Amount of photocatalyst: 0.5 g; Volume of reaction solution (H_2O): 1.0 L; Additive: 0.1 M $NaHCO_3$; CO_2 flow rate: 30 mL min^{-1}; Light source: 400 W high-pressure Hg lamp.

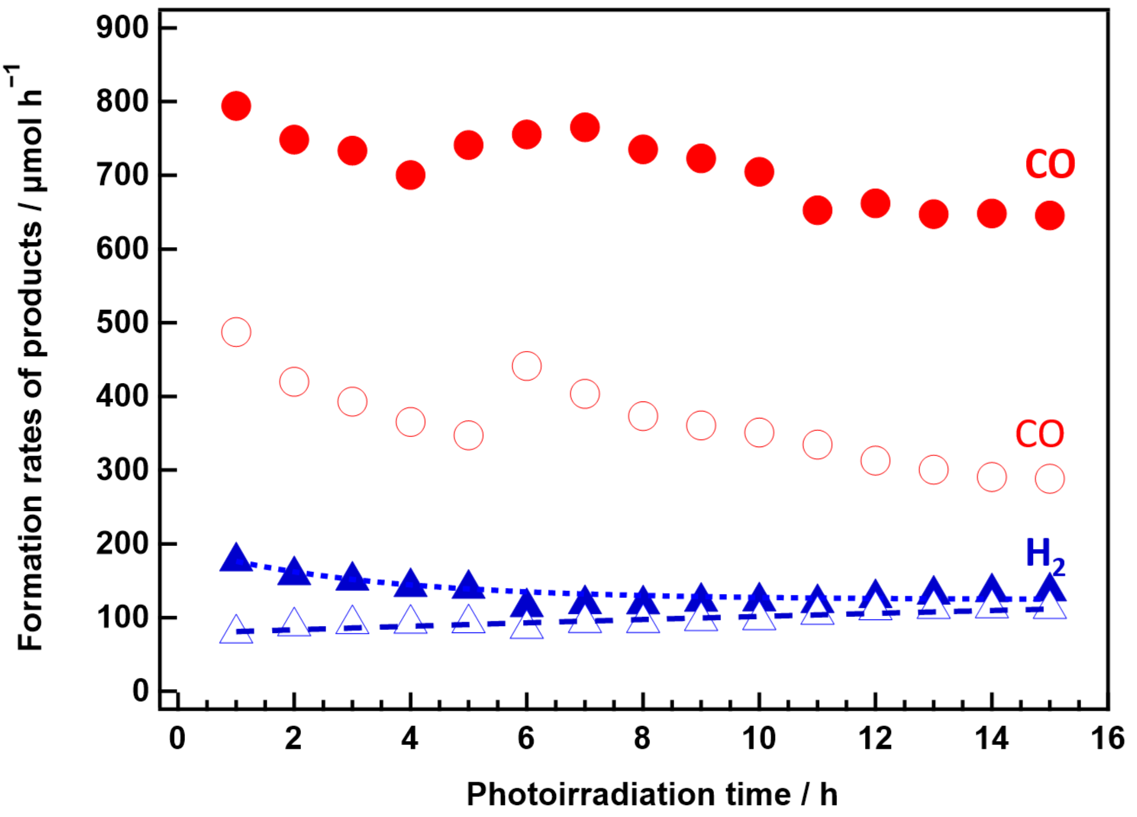

Supplementary Figure 2. **Product formation rates for 15 h.** Formation rates of CO (red circle) and H_2 (blue triangle) for the photocatalytic conversion of CO_2 by H_2O over Ag@Cr/Ga_2O_3 (hollow mark) and Ag@Cr/Ga_2O_3_Ca (solid mark).

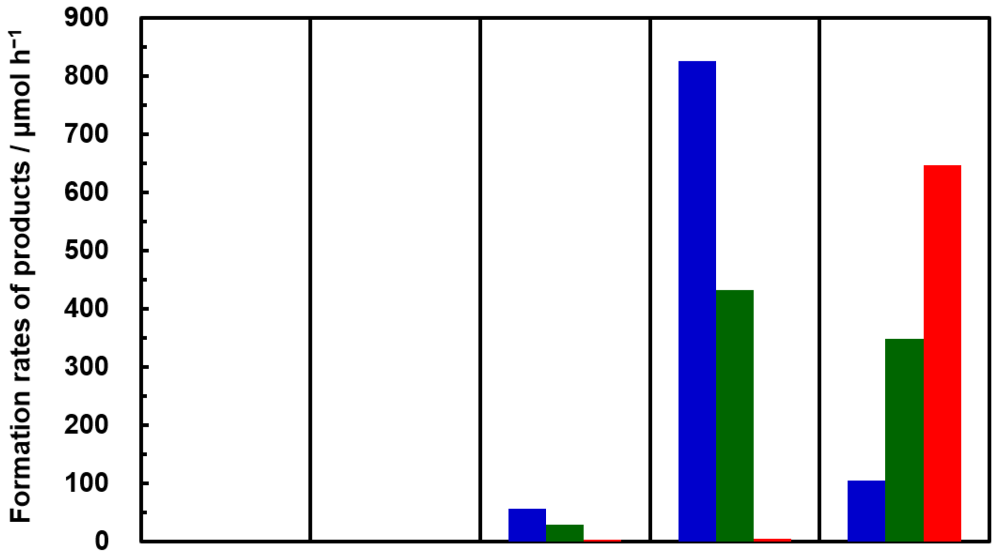

	1	2	3	4	5
Light	×	○	○	○	○
Photocatalyst	○	×	○	○	○
$NaHCO_3$	○	○	×	○	○
CO_2	○	○	○	×	○
all	○	○	○	○	○

Supplementary Figure 3. Various control experiments. Formation rates of H_2 (blue bars), O_2 (green bars), and CO (red bars) for the Ag-Cr/Ga_2O_3_Ca photocatalyst during photocatalytic conversion of CO_2. The data markers ○ and × indicate the presence and absence of each component, respectively. Amount of photocatalyst: 0.5 g; Volume of reaction solution (H_2O): 1.0 L; Additive: 0.1 M $NaHCO_3$; CO_2 flow rate: 30 mL min^{-1}; Light source: 400 W high-pressure Hg lamp.

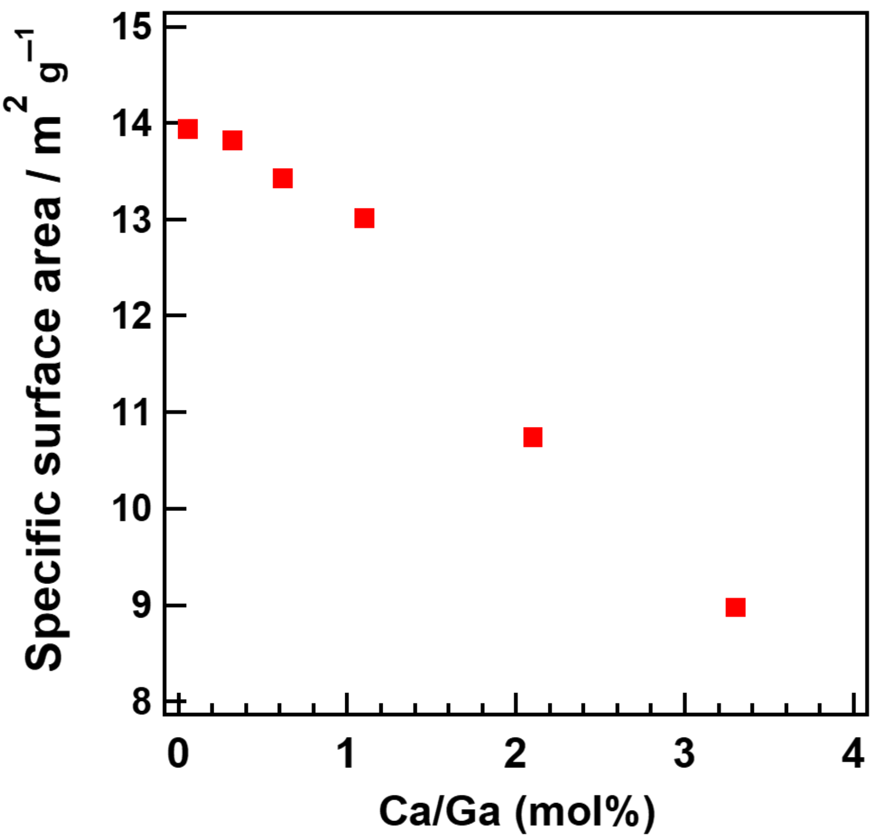

Supplementary Figure 4. BET specific surface areas. BET specific surface areas for Ga_2O_3_Ca_*x* with a Ca/Ga molar ratio *x* of 0.056, 0.32, 0.62, 1.1, 2.1, and 3.3 mol%.

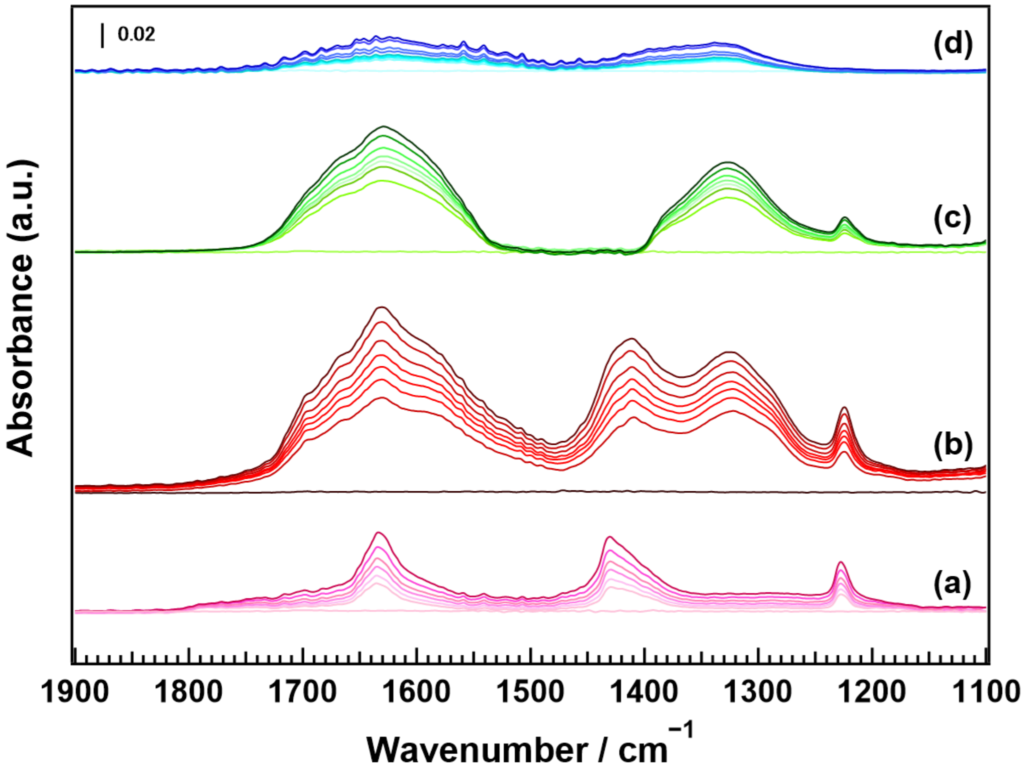

Supplementary Figure 5. FTIR spectra of CO_2 adsorption. CO_2 adsorbed on: (a) Ga_2O_3, (b) Ga_2O_3_Ca_1.1, (c) Ga_2O_3_Ca_3.3, and (d) $CaGa_4O_7$ after introducing the same amount of CO_2 at various pressures in the 0.1–40.0 Torr range.

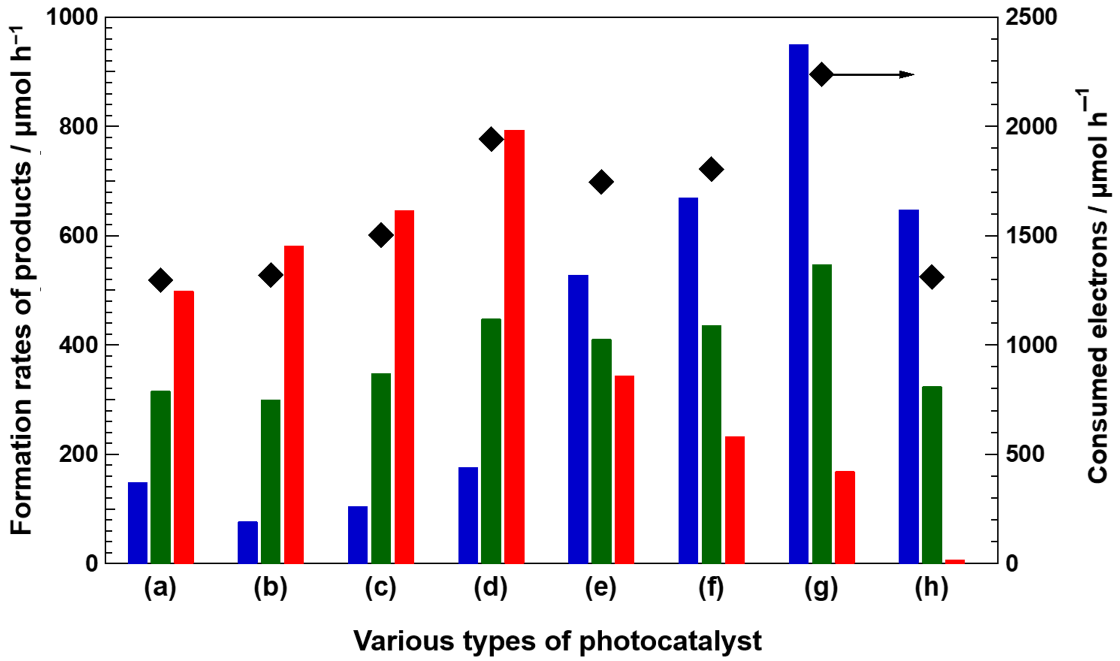

Supplementary Figure 6. Product formation rates and consumed electrons. Formation rates of H_2 (blue bars), O_2 (green bars), and CO (red bars), as well as the consumed electrons (black diamonds) for (a) Ag-Cr/Ga_2O_3, Ag-Cr/Ga_2O_3_Ca_x with a Ca/Ga molar ratio x of (b) 0.32 mol%, (c) 0.62 mol%, (d) 1.1 mol%, (e) 1.6 mol%, (f) 2.1 mol%, and (g) 3.3 mol%, and (h) Ag-Cr/$CaGa_4O_7$ during photocatalytic conversion of CO_2 by H_2O. Amount of photocatalyst: 0.5 g; Volume of reaction solution (H_2O): 1.0 L; Additive: 0.1 M $NaHCO_3$; CO_2 flow rate: 30 mL min^{-1}; Light source: 400 W high-pressure Hg lamp.

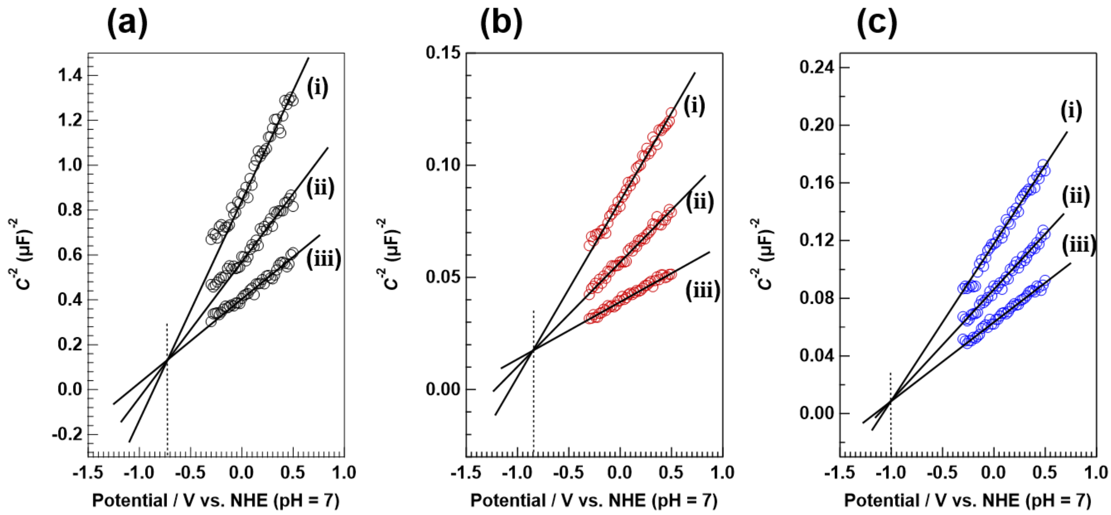

Supplementary Figure 7. Mott-Schottky plots of photocatalysts. Mott-Schottky plot for (a) Ga_2O_3/FTO, (b) Ga_2O_3_Ca_0.62/FTO, and (c) $CaGa_4O_7$/FTO based on the results of the impedance measurements at a frequency of (i) 39.8, (ii) 31.6, and (iii) 25.1 kHz. Electrolyte solution: Na_2SO_4 *aq*. (0.1 M, pH ca.7.0, Ag/AgCl), atmosphere: N_2.

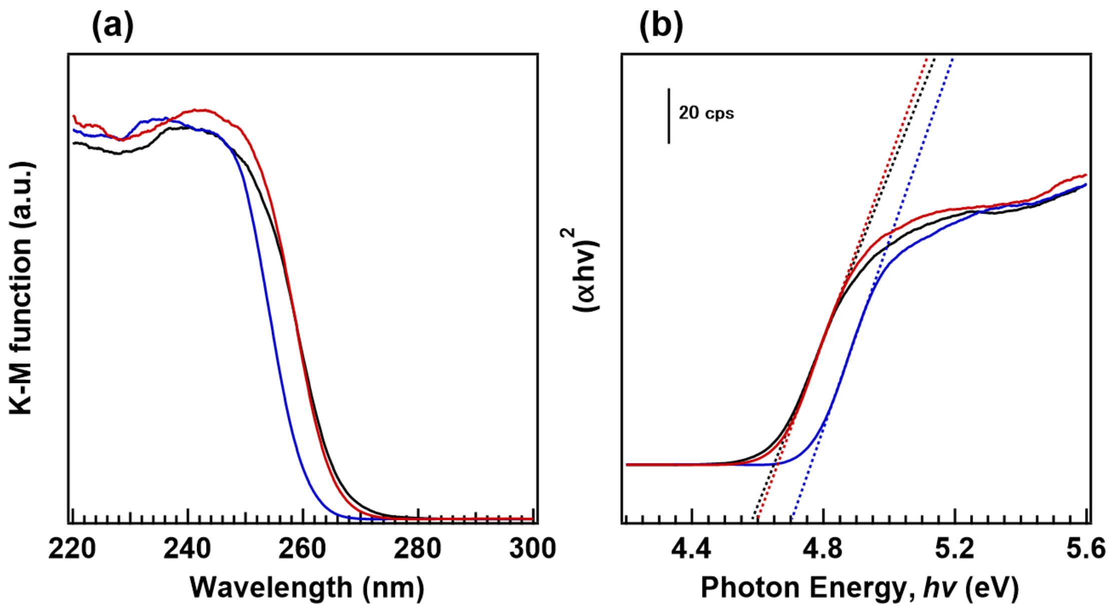

Supplementary Figure 8. Determination of band gap. (a) UV-visible spectra and (b) Davis-Mott plot presenting $(\alpha h\nu)^2$ versus photon energy for the determination of band gap of Ga_2O_3 (black line), Ga_2O_3_Ca_0.62 (red line), and $CaGa_4O_7$ (blue line).

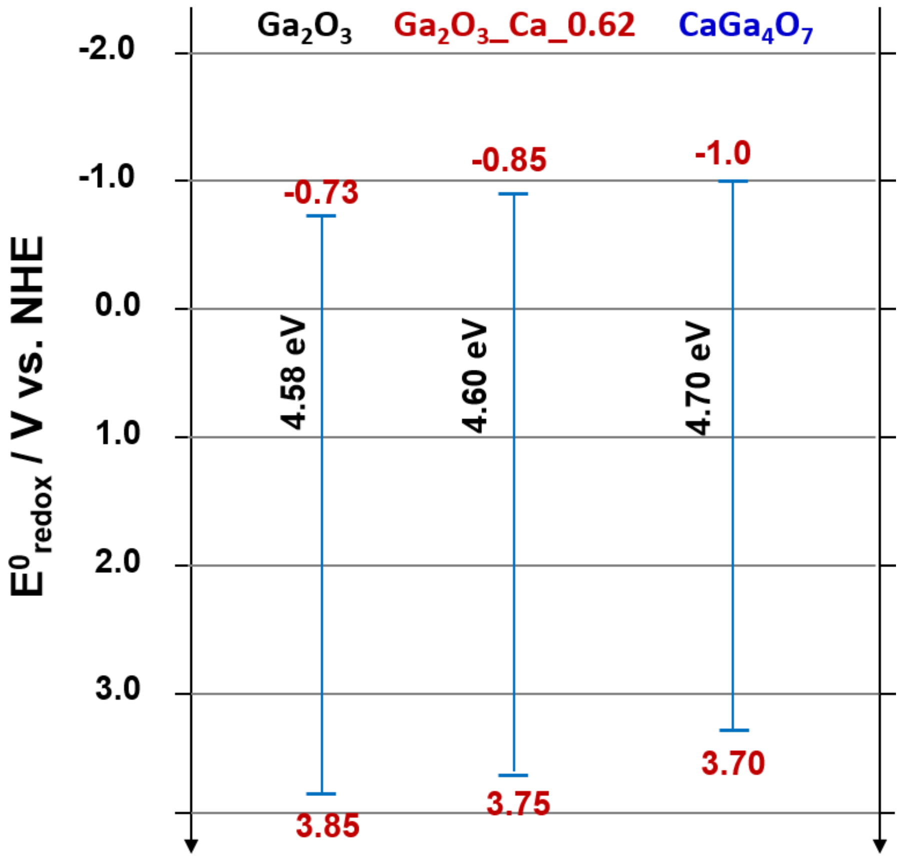

Supplementary Figure 9. Band positions of photocatalysts. Conduction band and valence band positions of Ga_2O_3, Ga_2O_3_Ca_0.62, and $CaGa_4O_7$.

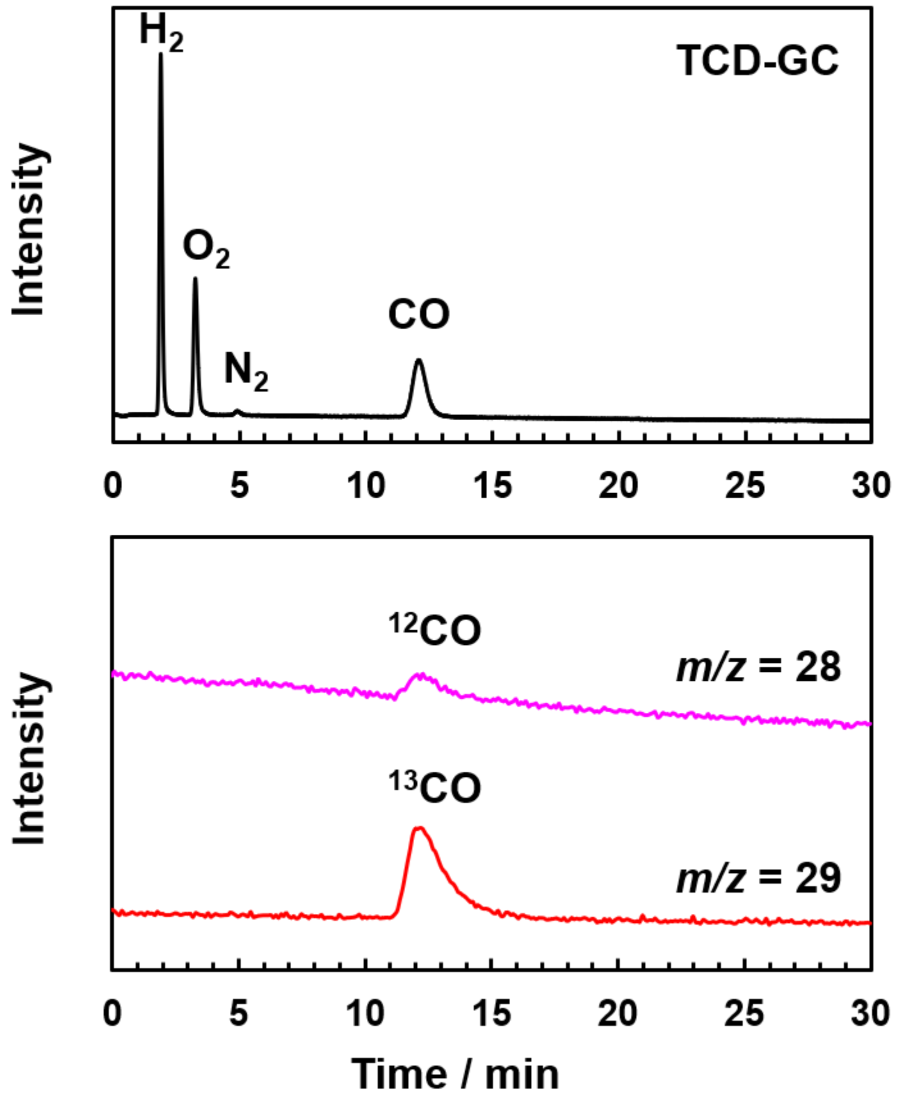

Supplementary Figure 10. Isotopic lead experiments. Gas chromatogram and mass spectra (m/z = 28 and 29) in the photocatalytic conversion of $^{13}CO_2$ by H_2O over the $CaGa_4O_7/Ga_2O_3$ photocatalyst physically mixed with 30 mol% of CaO with 1.0 mol% Ag-Cr as the cocatalyst.

Supplementary References

1 Iizuka, K., Wato, T., Miseki, Y., Saito, K. & Kudo, A. Photocatalytic reduction of carbon dioxide over Ag cocatalyst-loaded $ALa_4Ti_4O_{15}$ (A= Ca, Sr, and Ba) using water as a reducing reagent. *J. Am. Chem. Soc.* **133**, 20863-20868 (2011).

2 Nakanishi, H., Iizuka, K., Takayama, T., Iwase, A. & Kudo, A. Highly Active $NaTaO_3$-Based Photocatalysts for CO_2 Reduction to Form CO Using Water as the Electron Donor. *ChemSusChem* **10**, 112-118 (2017).

3 Anzai, A., Fukuo, N., Yamamoto, A. & Yoshida, H. Highly selective photocatalytic reduction of carbon dioxide with water over silver-loaded calcium titanate. *Catal. Commun.* **100**, 134-138 (2017).

4 Zhu, X., Anzai, A., Yamamoto, A. & Yoshida, H. Silver-loaded sodium titanate photocatalysts for selective reduction of carbon dioxide to carbon monoxide with water. *Appl. Catal. B* **243**, 47-56 (2019).

5 Wang, Z., Teramura, K., Hosokawa, S. & Tanaka, T. Photocatalytic conversion of CO_2 in water over Ag-modified $La_2Ti_2O_7$. *Appl. Catal. B* **163**, 241-247 (2015).

6 Wang, Z., Teramura, K., Hosokawa, S. & Tanaka, T. Highly efficient photocatalytic conversion of CO_2 into solid CO using H_2O as a reductant over Ag-modified $ZnGa_2O_4$. *J. Mater. Chem. A* **3**, 11313-11319, (2015).

7 Wang, Z. et al. Tuning the selectivity toward CO evolution in the photocatalytic conversion of CO_2 with H_2O through the modification of Ag-loaded Ga_2O_3 with a $ZnGa_2O_4$ layer. *Catal. Sci. Technol.* **6**, 1025-1032 (2016).

8 Teramura, K., Tatsumi, H., Wang, Z., Hosokawa, S. & Tanaka, T. Photocatalytic Conversion of CO_2 by H_2O over Ag-loaded SrO-modified Ta_2O_5. *Bull. Chem. Soc. Jpn.* **88**, 431-437 (2015).

9 Takayama, T., Iwase, A. & Kudo, A. Photocatalytic water splitting and CO_2 reduction over $KCaSrTa_5O_{15}$ nanorod prepared by a polymerized complex method. *Bull. Chem. Soc. Jpn.* **88**, 538-543 (2015).

10 Iguchi, S., Teramura, K., Hosokawa, S. & Tanaka, T. A $ZnTa_2O_6$ photocatalyst synthesized via solid state reaction for conversion of CO_2 into CO in water. *Catal. Sci. Technol.* **6**, 4978-4985 (2016).

11 Huang, Z., Teramura, K., Hosokawa, S. & Tanaka, T. Fabrication of well-shaped $Sr_2KTa_5O_{15}$ nanorods with a tetragonal tungsten bronze structure by a flux method for artificial photosynthesis. *Appl. Catal. B* **199**, 272-281 (2016).

12 Huang, Z., Teramura, K., Asakura, H., Hosokawa, S. & Tanaka, T. Flux method fabrication of potassium rare-earth tantalates for CO_2 photoreduction using H_2O as an electron donor. *Catal. Today* **300**, 173-182 (2018).

13 Huang, Z. et al. Sodium Cation Substitution in $Sr_2KTa_5O_{15}$ toward Enhancement of Photocatalytic Conversion of CO_2 Using H_2O as an Electron Donor. *ACS Omega* **2**, 8187-8197 (2017).

14 Pang, R., Teramura, K., Asakura, H., Hosokawa, S. & Tanaka, T. Highly selective photocatalytic conversion of CO_2 by water over Ag-loaded $SrNb_2O_6$ nanorods. *Appl. Catal. B* **218**, 770-778 (2017).

15 Iguchi, S. et al. Drastic improvement in the photocatalytic activity of Ga_2O_3 modified with Mg–Al layered double hydroxide for the conversion of CO_2 in water. *Sustain. Energy Fuels* **1**, 1740-1747 (2017).

16 Huang, Z., Teramura, K., Asakura, H., Hosokawa, S. & Tanaka, T. CO_2 capture, storage, and conversion using a praseodymium-modified Ga_2O_3 photocatalyst. *J. Mater. Chem. A* **5**, 19351-19357 (2017).

17 Tatsumi, H. et al. Enhancement of CO evolution by modification of Ga_2O_3 with rare-earth elements for the photocatalytic conversion of CO_2 by H_2O. *Langmuir* **33**, 13929-13935 (2017).

18 Pang, R. et al. Modification of Ga_2O_3 by an Ag–Cr core–shell cocatalyst enhances photocatalytic CO evolution for the conversion of CO_2 by H_2O. *Chem. Commun.* **54**, 1053-1056 (2018).
