# Supplementary material for: Enhanced CO evolution for photocatalytic conversion of CO2 by H2O over Ca modified Ga2O3
Source: Commun Chem. 2020 Oct 9;3:137. doi: 10.1038/s42004-020-00381-2 (PMC9814714; doi:10.1038/s42004-020-00381-2)
Supplement: Supplementary file 2 — Description of Additional Supplementary Files [file 42004_2020_381_MOESM2_ESM.pdf]

Description of Additional Supplementary Files

File Name: Supplementary Movie 1
Description: The formation of CO during the photocatalytic conversion of CO2 by H2O is directly manifested.
